# Editorial

**DOI:** 10.1007/s12592-020-00368-9

**Published:** 2020-12-21

**Authors:** Werner Thole, Karin Böllert, Karin Bock, Rita Braches-Chyrek, Bernd Dollinger, Catrin Heite, Fabian Kessl, Holger Ziegler

**Affiliations:** 1 Universität Kassel, Kassel, Deutschland; 2 Westfälische-Wilhelms-Universität Münster, Münster, Deutschland; 3 Technische Universität Dresden, Dresden, Deutschland; 4 Otto-Friedrich-Universität Bamberg, Bamberg, Deutschland; 5 Universität Siegen, Siegen, Deutschland; 6 Universität Zürich, Zürich, Schweiz; 7 Bergische Universität Wuppertal, Wuppertal, Deutschland; 8 Universität Bielefeld, Bielefeld, Deutschland

Vielleicht mehr als in den zurückliegenden fünf Jahrzehnten werden wir momentan angesichts der SARS-CoV-2-Pandemie damit konfrontiert, dass die „ganz alltäglichen Lebensbewältigungen und die ganz gewöhnlichen Lebensführungen […] zu einer selbst zu lösenden Herausforderung, zu einer selbst zu bewältigenden Lebensaufgabe, zu einer ungewissheitsbelasteten, riskanten sozialen Aufgabe“ werden können respektive sich entwickelt haben. Thomas Rauschenbach (1994, S. 91) formulierte diese Überlegungen aufgrund seiner Beobachtung, dass die gesellschaftlich vorgehaltenen und in den sozialen Milieus anzutreffenden und sich dort reproduzierenden Formen des Sozialen und der Solidarität zu implodieren scheinen. Menschen könnten infolgedessen das Soziale wie auch vertrauenswürdige, verlässliche Netzwerke der Solidarität nicht mehr durchgängig und umfänglich vorfinden und sind so aufgefordert und gezwungen, diese aktiv herzustellen. Die Suche nach verbindlichen Arrangements des Sozialen, auch um das Selbst sozial verordnen zu können, wird damit zu einer Angelegenheit der nach Sicherheit und Eingebundenheit strebenden Subjekte. Da dies jedoch nicht durchgängig gelingt, so die daran anschließende Idee, könnte sich die Soziale Arbeit angesprochen und herausgefordert fühlen, Praktiken der Solidarität zu inszenieren, die ermöglichen, die Strategien der Lebensführung wieder sozial zu rahmen. Die Beiträge im „Blickpunkt“ dieser Ausgabe der *Sozialen Passagen* präjudizieren eine andere Antwort. Nicht an die institutionelle Soziale Arbeit wird die Aufgabe adressiert, das Verschwinden des Sozialen, das Implodieren sozialen Zusammenhaltes und die Ausdünnung sozialer Sicherungssysteme auszugleichen, sondern anhand von theoretischen Überlegungen und illustriert über Beispiele wird dargestellt, dass soziale Bewegungen soziale wie sozialpolitisch initiierte Aushöhlungsprozesses des Sozialen auszugleichen in der Lage sind und zudem prägnanter und nachdrücklicher als die Soziale Arbeit politische Interventionen anzuregen vermögen – allerdings – noch – nicht in der Bundesrepublik Deutschland, sondern aktuell in Brasilien.

Der „Blickpunkt“ dieser Ausgabe widmet sich unter dem Titel „Zusammenhalt | diferença“ also Fragen, die die Möglichkeiten, gesellschaftlichen Zusammenhalt herzustellen, unter Beachtung sozialer Ungleichheiten und Differenz diskutieren. Dabei wird der Blick gerichtet auf Brasilien und eine Praxis, die sich wesentlich dokumentiert in den dortigen sozialen Bewegungen und den über diese initiierten Formen der Herstellung von sozialen Kontexten. Zusammengestellt und redigiert wurde der Themenschwerpunkt von *Benjamin Bunk* und *Emil Sobottka*. Für die sehr gelungene, inhaltlich sehr breit und interdisziplinär angelegte Komposition des Themenschwerpunktes möchten sich die Herausgeber*innen recht herzlich bei dem deutsch-brasilianischen Wissenschaftsduo bedanken. *Benjamin Bunk *und* Emil Sobottka* übernehmen es auch, mit ihrem Beitrag „Zusammenhalt und Differenz – Perspektiven auf Soziale Arbeit in Brasilien, oder: Zum Umgang mit Heterogenität dort, wo das Soziale extrem ungleich ist“ in den Schwerpunkt dieser Ausgabe der *Sozialen Passagen* einzuführen. In Reflexion der feinen Vereinnahmungen und Verschiebungen des Sozialen in der Bundesrepublik Deutschland wirft der Beitrag einen gesellschaftstheoretisch inspirierten Blick auf die Verfassung des Sozialen in Brasilien. Herausgebildet hat sich hier eine besondere Praxis des Umgangs und der Herstellung des Sozialen, die, nach der einseitigen, politischen Aufkündigung der Idee des Sozialen auf der Bundesebene, deutlicher als je zuvor darauf abzielt, kollektive Bewegungen, lokale Wissenskulturen und alternative, partiell auch tradierte solidarische Lebensweisen in urbanen wie ländlichen Räumen zu stärken. Der Beitrag thematisiert im Kern, wie es über eine Kritik der Verhältnisse gelingen kann, zu einer kritischen Praxis zu gelangen, und in welcher Form die bundesrepublikanischen Diskurse von den brasilianischen Thematisierungs- und Handlungspraxis angeregt werden können.

Mit der widerständigen Praxis von „Landnahmen, lokale Wissensordnungen und Widerständigkeiten in Amazonien“ beschäftigt sich *Maria Backhouse* in ihrem Beitrag. Widerständige Praktiken und lokale Wissensordnungen von Indigenen sind, so wird ausgeführt, in Brasilien seit Jahrhunderten eng mit Auseinandersetzungen um Landzugangs- und Landnutzungsrechte verknüpft. In der brasilianischen Verfassung sind die spezifischen Landzugangsrechte traditioneller Gemeinschaften seit den 1980er Jahren zwar garantiert, gegenwärtig werden diese Rechte allerdings von der Regierung des rechtsnationalen Präsidenten Jair Messias Bolsonaro attackiert und partiell eingeschränkt. Berichtet wird von neuen, sozialökologischen Initiativen einer auf Nachhaltigkeit setzenden Landeroberung. Daran anschließend diskutieren *Maria de Fátima Almeida Martins, Maria Isabel Antunes-Rocha *und* Geraldo Márcio Alves dos Santos* in ihrem Beitrag „Ländliche Erziehung und territoriales Engagement“ das Wissen und die Praktiken in der ländlichen Lehrer*innenbildung. Aufgegriffen werden Erfahrungen, die im Rahmen eines Projektes im Rahmen des Lehramtsstudiums Educação do Campo an der Bundesuniversität Minas Gerais gewonnen werden konnten. Gezeigt wird, dass ein Wechsel zwischen einem akademischen Umfeld und der alltäglichen Lebenswelt neue Bildungsprozesse bei Studierenden anzustoßen vermag. Unter dem Titel „Zwischen Territorien und lokalen Wissenspraktiken“ beschäftigen sich auch *Ana Maria R. Gomes, Shirley Aparecida de Miranda *und* Marina de Lima Tavares* mit der Praxis der Lehrer*innenqualifikation. Forderungen der Indigenen, ihre spezifischen Lebens- und Denkweisen beizubehalten, ihre eigenen Sprachen, Kulturen und Produktionsweisen zu erhalten und zu praktizieren sowie Forderungen nach der Tradierung indigenen Wissens und dessen Weitergabe motivieren die Entwicklung von interkulturellen Lehrer(innen)bildungen an brasilianischen Universitäten. Dargelegt wird, wie es auch mittels eines Lehrpraktikums gelingen kann, transformatorische Bildungsprozesse zu initiieren.

Wie Siedlungsabfälle als Ressource eine soziale wie nachhaltige Entwicklung befördern können, diskutieren *Francisco de Paula Antunes Lima, Marcelo Souza, Vivian Toffanelli, Viviane Zerlotini Silva, Fabiana Goulart de Oliveira *und* Juliana Gonçalvesi* in ihrem Aufsatz „Für einen neuen territorialen Stoffwechsel“. Die Autor*innen weisen darauf hin, dass Siedlungsabfälle erst im 20. Jahrhundert zu einer negativen Externalität, also zu Müll, wurden, während Abfall noch zu Beginn der Urbanisierungsprozesse eine begehrte Ressourcenquelle darstellte. Ausgehend von den Erfahrungen brasilianischer Müllsammler*innen mit wiederverwertbaren Materialien wird in dem Beitrag erörtert, wie ressourcenbewahrende Aktivitäten in Verbindung mit Ansätzen urbaner Agrarökologie zur Entstehung eines neuen Stoffwechsels zwischen Mensch und Natur beitragen und die Implementierung einer nachhaltigen, solidarischen Ökonomie fördern. Wie soziale Projekte der solidarischen Ökonomie Formen der kapitalistischen Produktionsweise infrage stellen können, illustrieren auch *Sibelle Cornélio Diniz, Bruno Siqueira Fernandes* und *Roberto Luís Monte-Mór* in ihrem Aufsatz „Soziale solidarische Ökonomie in dekolonialer Hinsicht?“. In der kapitalistischen Peripherie des Globalen Südens, so führen die Autor*innen aus, hat die Debatte zu den kolonialen Erfahrungen gegenwärtig eine besondere Bedeutung. In diesem Artikel wird argumentiert, dass die kapitalistischen und kolonialen Beziehungen im Globalen Süden spezifische Bedingungen für alternative, soziale wie solidarische Ökonomien anregen können.

In der Rubrik „Forum“ diskutiert *Markus Sauerwein* die „Konturen einer Weiterentwicklung der Didaktik für Sozialpädagogik“. Seine Diagnose von zunehmenden Scholarisierungsprozessen in sozialpädagogischen Arbeitsfeldern und einer damit einhergehenden, verstärkten Kompetenzorientierung bilden Basis für die vorgetragene Ausgangsmarkierung, dass nachdrücklicher als bislang Fragen der sozialpädagogischen Didaktik diskutiert werden sollten. Eine Durchsicht des gegenwärtigen Diskussionsstandes zeigt, so führt der Autor aus, dass zwar Unterrichtsprinzipien und Lehrmethoden einer sozialpädagogischen Didaktik benannt werden, es jedoch an einer hierauf bezogenen Qualitätsdiskussion fehlt. Der Beitrag knüpft an dieser Feststellung an und argumentiert, dass Kompetenzorientierung in Bezug auf die fachschulische Ausbildung von pädagogischen Fachkräften zwar durchaus kritisch diskutiert werden kann, letztlich jedoch nicht ignoriert bleiben sollte. Über diese Beobachtung sieht sich der Autor motiviert, zu empfehlen, das fachschulische Qualifikationscurriculum stärker kompetenzorientiert auszurichten. „Freiraum im Jugendalter – ein theoretischer und empirischer Versuch“ ist der zweite Beitrag von *Katrin Peyerl *und* Ivo Züchner* in der Rubrik „Forum“ betitelt. Vorgestellt wird ein theoretisches Modell, das Freiraum über die Dimensionen Raum, Zeit und Selbstbestimmung zu konzipieren und anhand einer quantitativen Studie zum Aufwachsen Jugendlicher in Internaten zu operationalisieren versucht.

*Margarete Kilian *und* Moritz Rinn* stellen in ihrem Beitrag „Aufsuchende Soziale Arbeit in Konflikten um städtische Räume“ die Ergebnisse eines explorativen Forschungsprojekts vor. Ausgangspunkt der Studie war die Annahme, dass Akteur*innen stadtteilbezogener Sozialer Arbeit in Aneignungskonflikten, in die Adressat*innen involviert sind, über einen kontextspezifischen Handlungsspielraum verfügen, in dem Ziele definiert, Interventionsanlässe gewählt, handlungsleitende Problematisierungen hervorgebracht und angeeignet werden können. Die Befunde der Studie zeigen ein Spektrum unterschiedlicher sozialarbeiterischer Vorgehensweisen an, die von parteilichen Interventionen und Konfliktorientierungen bis zur Übernahme externer Problematisierungen und aktiven Beteiligungen an einer „dislocation“ problematisierter Personen reichen. Mit den „Akteur*innen Sozialer Arbeit in der Radikalisierungsprävention“ beschäftigen sich *Carmen Figlestahler *und* Katja Schau* in ihrem Aufsatz. Diskutiert wird der spezielle Bereich der Präventions- und Ausstiegsarbeit im Feld des demokratiefeindlichen Islamismus. Anhand einer qualitativen Fallstudie zum regionalen Kooperationsarrangement wird die multiprofessionelle Problembearbeitung von demokratiefeindlichem Islamismus untersucht und damit der Frage nachgegangen, wie Informationsweitergaben und Grundprinzipien der Arbeit zwischen sicherheitsbehördlichen und sozialpädagogischen Akteur*innen ausgehandelt werden können.

Im „Zwischenruf“ berichten *Lucia Scalco *und* Emil Sobottka *unter der Überschrift „Möge die Solidarität ansteckender sein als das Virus!“ von einer Aktion, die eine ressourcenärmere Gemeinde am Rande der brasilianischen Stadt Porto Alegre entwickelt hat. Berichtet wird, wie sich in der Gemeinde Morro da Cruz ein solidarisches Zugehörigkeitsgefühl über Formen interner Selbstorganisation und eine angehende Vertrautheit mit neuen Technologien entwickeln konnten. Ausgehend von diesen solidarischen, das Soziale fördernden Erfahrungen entstand eine Sofortmaßnahme, welche in den ersten Monaten der Quarantäne im Zuge der Corona-Pandemie durchgeführt wurde.

Eine Möglichkeit, in das Berufsfeld der Sozialen Arbeit einzumünden, besteht darin, einen Fernstudiengang erfolgreich zu absolvieren. Der Beitrag „Der Lizenzierungsweg Fernstudium in der Sozialen Arbeit: Der Boom des großen Unbekannten“ von *Nikolaus Meyer *und* Christina Buschle* in der Rubrik „Praxis Hochschule“ beleuchtet und diskutiert die Entwicklung der Studierendenzahlen in sozialpädagogischen Fernstudienprogrammen. Kritisch erörtert wird die quantitativ wachsende Bedeutung des Fernstudiums für den Lizenzierungsprozess zukünftiger Beschäftigter in Handlungsfeldern der Sozialen Arbeit.

In den „Forschungsnotizen“ werden aktuelle Forschungsprojekte vorgestellt. *Martina Koch, Markus Steffen *und* Rahel Bühler* stellen ihr qualitatives Forschungsvorhaben „Hausbesuche im Kindes- und Erwachsenenschutz in der Schweiz“ vor. Zwei Dissertationsvorhaben werden präsentiert: *Jens Vogler* skizziert die Anlage seines Vorhabens „Arbeitsbündnisse in der Migrationsgesellschaft. Professionelle und zivilgesellschaftliche Beratung in Handlungsfeldern Sozialer Arbeit“ und *Melanie Rühmling* stellt ihre Studie „Lebensverhältnisse in ländlichen Räumen – Ein Dissertationsprojekt über das Bleiben von Frauen in ländlichen Räumen“ vor. Das Forschungsprojekt zu „Sozialraumorientierter Schulsozialarbeit an Sekundarschulen in Baden-Württemberg“ von *Mirjana Zipperle, Katharina Meier *und* Andreas Karl Gschwind* komplettiert diese zweite Ausgabe der Sozialen Passagen 2020.

*

Betroffen und mit tiefster Trauer mussten die Herausgeber*innen den Tod von Prof. Dr. Dr. h. c. mult. Hans-Uwe Otto zur Kenntnis nehmen. Das Herausgeber*innenkollektiv der *Sozialen Passagen *war mit Hans-Uwe Otto in unterschiedlichster Form, als Freund, Kollege, wissenschaftlichem Diskurspartner oder Doktorvater, eng verbunden. Gemeinsam schätzen wir sein wissenschaftliches Erkenntnisinteresse, seine theoretischen Initiativen und sein bis zum Tod andauerndes Bemühen, die Wirklichkeit sozialpädagogischen Handelns, sozialpolitischer Gegenwartsgestaltungen sowie die Bedingungen des Aufwachsens von Kindern und Jugendlichen auch empirisch aufzuklären. Und wie kein anderer in der sozialpädagogischen Community thematisierte er die Professionalität und die Professionalisierungsbedürftigkeit der Sozialen Arbeit unter kapitalistischen Produktionsbedingungen. Das Projekt der *Sozialen Passagen* begleitete er skeptisch und stets kritisch, aber durchgehend anerkennend und die mit dem Projekt verbundenen Intentionen, die interdisziplinären Blicke auf die Soziale Arbeit theoretisch und empirisch klarer zu zeichnen und schärfer zu fassen, immer würdigend.

Mit Hans-Uwe Otto teilten wir weiterhin das Anliegen, das gesellschaftlich Gegebene als eine veränderbare Wirklichkeit anzusehen, die kritisch zu diskutieren wir herausgefordert sind. Als aufmerksamen Wegbegleiter und zuweilen scharfzüngigen Kritiker der *Sozialen Passagen *werden wir Hans-Uwe Otto vermissen. Seiner Familie und seinen engen Freund*innen gilt unser tiefstes Mitgefühl. Deine optimistische Haltung, dein Credo, dass die gegenwärtig erlebte Gesellschaft einen Zwischenzustand dokumentiert und für ihre bessere Gestaltung lediglich ein neuer Entwurf anzufertigen ist, wird uns weiterhin anregen, Hans-Uwe Otto.

*
